# Endoscopic therapies for patients with obesity: a systematic review and meta-analysis

**DOI:** 10.1007/s00464-023-10390-6

**Published:** 2023-09-20

**Authors:** Zachary N. Weitzner, Jennifer Phan, Meron M. Begashaw, Selene S. Mak, Marika S. Booth, Paul G. Shekelle, Melinda Maggard-Gibbons, Mark D. Girgis

**Affiliations:** 1 Department of Surgery, David Geffen School of Medicine, University of California Los Angeles, 757 Westwood Plaza, Los Angeles, CA 90095 USA; 2 Department of Gastroenterology, Keck School of Medicine, University of Southern California, Los Angeles, USA; 3 Veterans Health Administration, Greater Los Angeles Healthcare System, Los Angeles, USA; 4 RAND Corporation, Santa Monica, USA

**Keywords:** Bariatric endoscopy, Intragastric balloon, Endoscopic sleeve gastrectomy, AspireAssist

## Abstract

**Background:**

Obesity is a major threat to public health and traditional bariatric surgery continues to have low utilization. Endoscopic treatments for obesity have emerged that offer less risk, but questions remain regarding efficacy, durability, and safety. We compared the efficacy of endoscopic bariatric procedures as compared to other existing treatments.

**Methods:**

A literature search of Embase, Cochrane Central, and Pubmed was conducted from January 1, 2014 to December 7, 2021, including endoscopic bariatric therapies that were FDA or CE approved at the time of search to non-endoscopic treatments. Thirty-seven studies involving 15,639 patients were included. Primary outcomes included % total body weight loss (%TBWL), % excess body weight loss (%EBWL), and adverse events. Secondary outcomes included quality of life data and differences in hemoglobin A1C levels. Strength of clinical trial and observational data were graded according to the Cochrane methods.

**Results:**

Intragastric balloons achieved greater %TBWL with a range of 7.6–14.1% compared to 3.3–6.7% with lifestyle modification at 6 months, and 7.5–14.0% compared to 3.1–7.9%, respectively, at 12 months. When endoscopic sleeve gastroplasty (ESG) was compared to laparoscopic sleeve gastrectomy (LSG), ESG had less %TBWL at 4.7–14.4% compared to 18.8–26.5% after LSG at 6 months, and 4.5–18.6% as compared to 28.4–29.3%, respectively, at 12 months. For the AspireAssist, there was greater %TBWL with aspiration therapy compared to lifestyle modification at 12 months, 12.1–18.3% TBWL versus 3.5–5.9% TBWL, respectively. All endoscopic interventions had higher adverse events rates compared to lifestyle modification.

**Conclusion:**

This review is the first to evaluate various endoscopic bariatric therapies using only RCTs and observational studies for evaluation of weight loss compared with conservative management, lifestyle modification, and bariatric surgery. Endoscopic therapies result in greater weight loss compared to lifestyle modification, but not as much as bariatric surgery. Endoscopic therapies may be beneficial as an alternative to bariatric surgery.

**Supplementary Information:**

The online version contains supplementary material available at 10.1007/s00464-023-10390-6.

Obesity continues to be a major health concern in the US. In 2020, an estimated 41.9% of the US population was obese with 9.2% diagnosed as severely obese [1, 2]. While the prevalence is high, there are a range of therapeutic options, including diet modifications, medications, endoscopic therapies, and surgery. Historically, surgery is the most effective option, generating weight loss of up to 70% excess body weight loss (%EBWL) and 30% total body weight loss (%TBWL) [3]. However, less than 1% who qualify undergo operative intervention. Barriers to patients undergoing bariatric surgery include fear of surgical risk and poor access to healthcare [4].

In response, many endoscopic modalities for treating obesity have emerged that potentially offer less risk, are less costly, and possibly more accessible to patients and providers. These include intragastric balloons, endoscopic sleeve techniques, and other novel therapies. Although numerous reviews address individual endoscopic therapies or focus on weight loss, a comprehensive review addressing the variety of endoscopic therapies compared to non-pharmacologic medical therapy (lifestyle therapy), pharmacologic therapy, and surgical options in regards to not only weight loss, but also complications and safety has not been performed. Thus we conducted a systematic review and meta-analysis to assess the effectiveness of endoscopic bariatric therapies on weight loss, morbidity, mortality, and comorbid conditions compared to non-endoscopic treatments of obesity.

## Methods/literature search

### Search strategy

This systematic review was reported according to PRISMA guidelines and registered in PROSPERO, #CRD42021270205 (Endoscopic Bariatric Therapies: A Systematic Review). This review is derived and updated from a report funded by and prepared for the Department of Veterans Affairs [5].

### Search strategy

Pubmed, Embase, and Cochrane Central were searched for broad terms relating to “gastric balloon”, “bariatric endoscopic procedure”, and “endoscopic gastroplasty”. The search was conducted on January 23, 2022 for studies published between January 1, 2014 and January 23, 2022. Studies published prior to 2014 would have been based on data from procedures done in 2012 or earlier, which we considered to be not as relevant to practice today. See Online Appendix A for complete search strategy. Our search strategy included studies published in English only. References of identified articles were searched for additional relevant articles.

### Study selection

As our sponsor was the Department of Veterans Affairs, only therapies that were FDA or CE approved for use or were undergoing trials nearing approval were considered. These criteria and therpies were agreed upon by an expert panel in the field of bariatric endoscopy. Approved procedures included intragastric balloon (IGB, including ReShape Duo™, Obalon™, Spatz3™, Orbera™, Elipse™), endoscopic sleeve gastroplasty (ESG), primary obesity surgery endoluminal (POSE) and AspireAssist™. Included studies had to compare an approved endoscopic bariatric therapy to one or more alternate bariatric therapies (lifestyle, pharmaceutical, or surgical). Randomized clinical trials and observational studies were included. Case series with sample size greater than 500 were included for adverse event data only. Studies had to have at least 6 months of follow-up to be included. Studies needed to report weight loss as an outcome. We excluded studies evaluating bariatric procedures as bridging therapy and when endoscopic procedures were compared to each other, such as varying endoscopic balloon sizes or primary obesity surgery endoluminal versus endoscopic sleeve gastroplasty. Studies which did not clearly compare one therapy to another, such as those that compared endoscopic bariatric therapies with pharmacologic therapy to pharmacologic therapy alone, were excluded. Studies were screened independently by two reviewers to ensure they satisfied these criteria, reconciling disagreements via group discussion.

ESG and POSE trials were included together under endoscopic gastric plications (GP) given the similarity in endoscopic technique.

### Data abstraction

The primary effectiveness outcome assessed was weight loss, reported as percent total body weight loss (%TBWL) and percent excess body weight loss (%EBWL). Excess body weight loss was defined as the difference in pre-treatment weight and post-treatment weight divided by the difference between pre-treatment weight and ideal body weight, described as a percentage. Secondary effectiveness outcomes included quality of life (QoL) and hemoglobin A1c (HbA1c). Outcome data, along with reports of adverse events, and study design, participant demographics, and data required for quality assessment were extracted independently by two coauthors and recorded in a standardized electronic data collection sheet. Outcomes were assessed at 6 months (or between 4 and 8 months), 12 months (or between 9 and 16 months), and thereafter annual time periods.

### Quality assessment/risk of bias

The risk of bias of included studies was assessed using the Cochrane Risk of Bias tool for randomized studies and the Cochrane Risk of Bias in Non-Randomized Studies of Interventions (ROBINS-I) tools by 2 authors independently, with discrepancies reconciled after joint review of articles and discussion.

### Data synthesis

For comparisons with at least 3 studies of the same intervention and similar patient populations and outcomes, meta-analyses were performed. Endoscopic sleeve gastroplasty and POSE trials were included together under endoscopic gastric plications (GP) given the similarity in endoscopic technique. Pooled estimates of effect were reported as mean differences (MD) with their 95% confidence intervals (CI). The potential for heterogeneity of treatment effects was tested by *I*^2^. The presence of possible publication bias was evaluated using the Begg rank correlation and Egger regression tests. *p*-values less than 0.05 were considered statistically significant. A narrative analysis was performed for the remainder of outcomes. Continuous outcomes were analyzed using the mean or median along with a measure of dispersion (standard deviation, interquartile range) to calculate the difference and 95% CI between arms. For binary outcomes, the risk difference was reported with 95% CI. Data were stored and displayed using Microsoft Excel® (Microsoft, Redmond, WA) and R (R Core Team).

### Rating the body of evidence

The Grading of Recommendations Assessment, Development, and Evaluation (GRADE) working group criteria was used to assess the certainty of evidence across studies based on risk of bias, imprecision, inconsistency, indirectness, and publication bias [6]. The GRADE system results in a rating of high, moderate, low, or very low certainty of evidence.

### Role of the funder

The funder, the Department of Veterans Affairs, was involved in setting the scope of the review and was a peer reviewer of the draft report on which this manuscript is based, but otherwise did not participate in the identification and analysis of data or the decision to publish.

## Results

A total of 3541 citations were identified by the search and assessed for eligibility. After title, abstract, and full-text screening for eligibility criteria, 36 full-text articles were included (16 RCTs, 14 observational trials, 6 case series, see PRISMA flow chart in Online Appendix B). A total of 15 studies comparing IGB to lifestyle therapy [7–21], 3 studies comparing ESG to lifestyle therapy [22–24], 7 studies comparing ESG to laparoscopic sleeve gastrectomy (LSG) [25–31], and 1 study comparing ESG to adjustable gastric band (AGB) [25]. There were 2 studies comparing AspireAssist to lifestyle [32, 33] and 1 study comparing AspireAssist to Roux-Y gastric bypass [34]. No studies were identified comparing any EBT directly to pharmacologic weight loss therapies. These studies enrolled a total of 15,639 patients, of which the mean age was 42.3 years, 82.2% were female, and average BMI was 41.15 kg/m^2^. Tables 1, 2, and Appendices C–G summarize the included studies’ findings and risk of bias assessments.

**Table 1:** Adverse events

Study, year design	Total complication	30-day readmission	30-day reintervention	Nausea	Vomiting	Dehydration	Abdominal pain	Gastric ulceration	Bleeding	GERD
*IGB vs lifestyle*										
Sullivan 2018 RCT	Risk diff (CI) 0.25 (0.17, 0.32)			0.38 (0.29, 0.46)	0.07 (0.01, 0.14)	− 0.01 (− 0.02, 0.01)	0.49 (0.41, 0.58)	0.01 (0, 0.01)	0.06 (0.03, 0.09)	
Courcoulas 2017 RCT	0.27 (0.19, 0.35)	7.5		0.82 (0.75, 0.88)	0.70 (0.62, 0.78)	0.14 (0.09, 0.2)	0.54 (0.45, 0.62)			0.25 (0.17, 0.33)
Abu Dayyeh 2021 RCT	0.03 (0, 0.06)		0.00 (0, 0)	0.90 (0.86, 0.95)	0.71 (0.65, 0.78)	0.02 (0, 0.04)	0.56 (0.49, 0.63)	0.02 (0, 0.04)		
Ponce 2012 RCT	0.33 (0.13, 0.53)									
Ponce 2015 RCT	0.57 (0.5, 0.64)							0.35 (0.28, 0.42)		
Fuller 2013 RCT				0.72 (0.55, 0.89)	0.69 (0.51, 0.86)	0.26 (0.1, 0.41)	0.56 (0.37, 0.74)			
Moore 2019 Case series	14.3%			63%	31%	0.07%	5.29%	0.82%		0.82%
Sander 2017 Case series									0.01%	
Mathus-Vliegen 2015 Case series	5.9%		0.2%			0.2%		0.9%	0.6%	
*ESG vs lifestyle*										
Sullivan 2017 RCT	0.09 (0.01, 0.17)			0.14 (0.07, 0.21)	0.19 (0.13, 0.24)				0.01 (0, 0.01)	0.04 (− 0.02, 0.09)
Cheskin 2020 Observ	0.05 (0.01, 0.09)					0.01 (− 0.01, 0.03)		0.03 (0, 0.06)		
Alqahtani 2019 Case series		2.4%		92.4%					0.7%	
*AspireAssist vs lifestyle*										
Thompson 2017 RCT	0.78 (0.71, 0.86)		0.01 (− 0.01, 0.03)		0.17 (0.1, 0.24)		0.38 (0.29, 0.47)		0.02 (− 0.01, 0.04)	
*ESG vs LSG*										
Fayad 2019 Observ	− 0.12 (− 0.23, 0)	− 0.06 (− 0.18, 0.07)	0.00 (0, 0)		0.01 (0.01, 0.16)	− 0.01 (− 0.04, 0.01)			− 0.03 (− 0.07, 0.01)	− 0.13 (− 0.22, − 0.03)
Fiorillo 2020 Observ	− 0.04 (− 0.13, 0.04)	− 0.04 (− 0.13, 0.04)							− 0.04 (− 0.13, 0.04)	− 0.30 (− 0.49, − 0.12)
Lopez-Nava 2021 Observ	− 0.04 (− 0.1, 0.01)								− 0.04 (− 0.1, 0.01)	
Novikov 2018 Observ	− 0.07 (− 0.13, − 0.01)	− 0.02 (− 0.04, 0.01)	− 0.04 (− 0.09, − 0.01)		− 0.01 (− 0.02, 0.01)					

Data are presented as mean difference between comparative arms when appropriate, where positive values favor endoscopic therapy and negative values favor comparative arm. For case series, complication data are presented in percentages

*ESG* endoscopic sleeve gastroplasty, *IGB* intragastric balloon, *LSG* laparoscopic sleeve gastrectomy

**Table 2:** Hemoglobin A1c and quality of life outcomes for bariatric endoscopic procedures compared to lifestyle or surgery

	HbA1c	Quality of life
	Negative is better for endoscopic treatment	Positive is better for endoscopic treatment
	MD [95% CI]	MD [95% CI]
IGB vs lifestyle		
Abu Dayyeh, 2021, 9 months (RCT)	− 0.73 [− 1.49, 0.02]^a^	
Sullivan 2018, 6 months (RCT)	0.00 [− 0.14, 0.14]	
Courcoulas 2017, 6 months (RCT)		7.5 [NR]
Courcoulas 2017, 12 months (RCT)		6.40 [NR]
ESG vs lifestyle		
Ahmed 2019, 6 months (RCT)		0.13 [0.02, 0.231]^c^
Sullivan 2017, 6 months (RCT)	− 0.03 [− 0.9, 0.04]	
Sullivan 2017, 12 months (RCT)	− 0.03 [− 0.11, 0.05]	2.9 [1.6, 4.2]
AspireAssist vs lifestyle		
Thompson 2017, 12 months (RCT)	− 0.14 [NR]	
ESG vs LSG		
Benias 2020, 12 months (Obs)	− 7.7 [− 11.0, − 4.8]^b^	
Fiorillo 2020, 6 months (Obs)		1.00 [− 8.6, 10.64]
Sadek 2017, 6 months (Obs)		7.9 [NR]

*ESG* endoscopic sleeve gastroplasty, *IGB* intragastric balloon, *LSG* laparoscopic sleeve gastrectomy

^a^Only among those with type 2 diabetes and baseline HbA1c > 7.5%

^b^Mean difference percent change from baseline

^c^Risk difference (different between percent reporting high QOL)

### Study quality/risk of bias

Among these RCTs, the most common sources of bias were lack of blinding and incomplete outcome data. All randomized controlled trials had high risk of bias in at least one domain of the Cochrane Risk of Bias Tool (citation: https://methods.cochrane.org/bias/resources/rob-2-revised-cochrane-risk-bias-tool-randomized-trials). Among the observational studies, the most common sources of bias were confounding, selection of participants, missing data, and measurement of outcomes. All observational studies but one had unknown or high risk of bias in at least one domain.

### Intragastric balloon

#### Weight loss

**Fig. 1:**
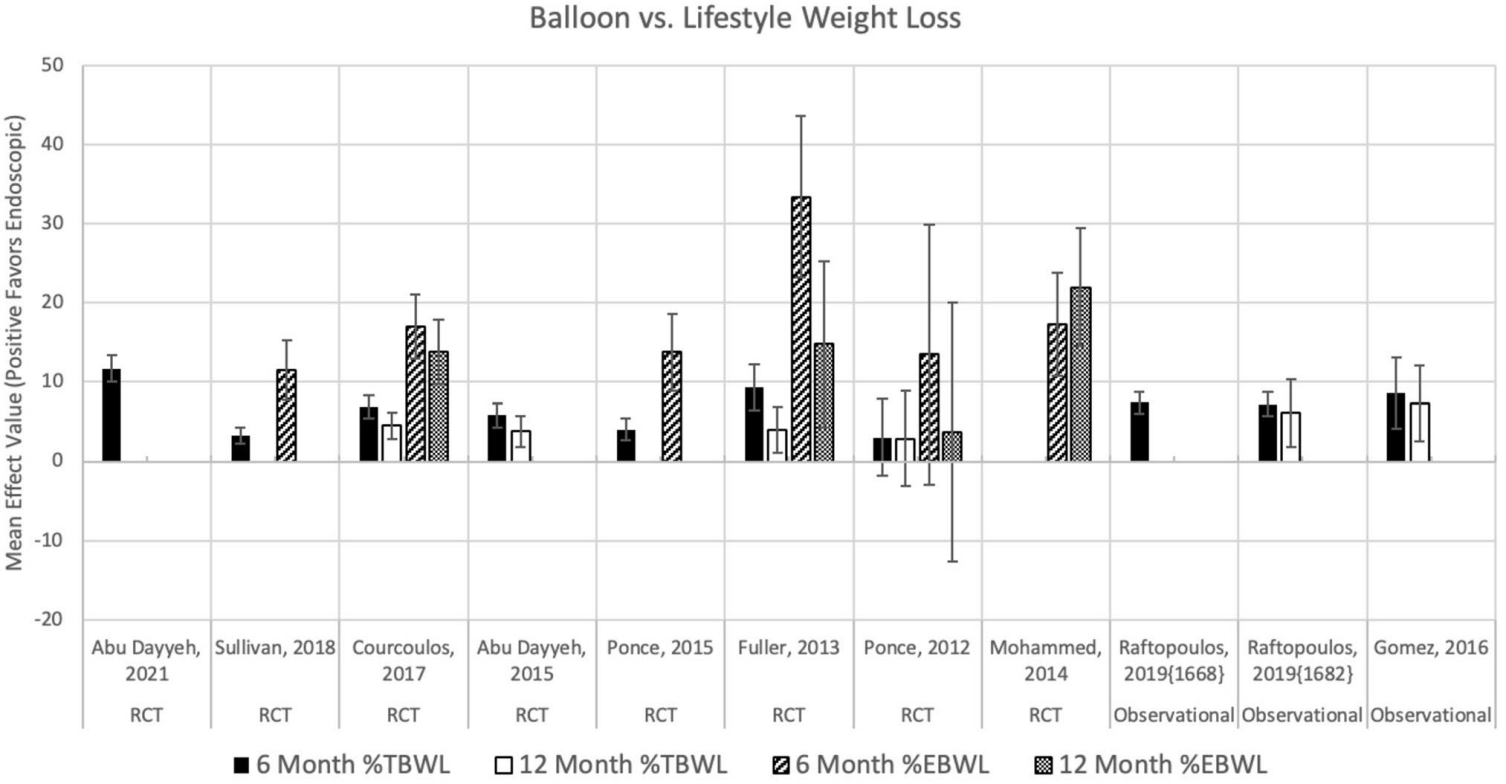
Weight loss outcomes for balloon versus lifestyle at 6 and 12 months. Of note, the study that failed to demonstrate significance in %EBWL or %TBWL at 6 and 12 months compared to lifestyle therapy evaluated the ReShape Duo two balloon system, which was not evaluated in any other RCTs

Eleven studies were identified comparing IGB to lifestyle modification using %TBWL as the weight loss outcome [7–16] (Fig. 1). Eight studies were randomized trials and 3 studies were observational. Ten studies reported 6 month %TBWL outcomes, and in 9 of these studies IGB was associated with greater %TBWL than lifestyle modification (range of 7.6–14.1% compared to 3.3–6.7% with lifestyle modification). Six studies reported 12 month %TBWL outcomes, and in five of these studies IGB was associated with greater %TBWL (range of 7.5–14.0% after IGB compared to 3.1–7.9% with lifestyle modification [7–9, 12, 14, 15]). The 4 RCTs reporting %TBWL at 12 months were pooled into a meta-analysis, with a random effect pooled estimate of 4.1% TBWL (95% CI, 3.0%–5.3%). Six studies and 4 studies, respectively, reported 6 and 12 month outcomes as %EBWL, and 5 (of 6) and 3 (of 4) studies found IGB was associated with greater weight loss than lifestyle modification.

**Fig. 2:**
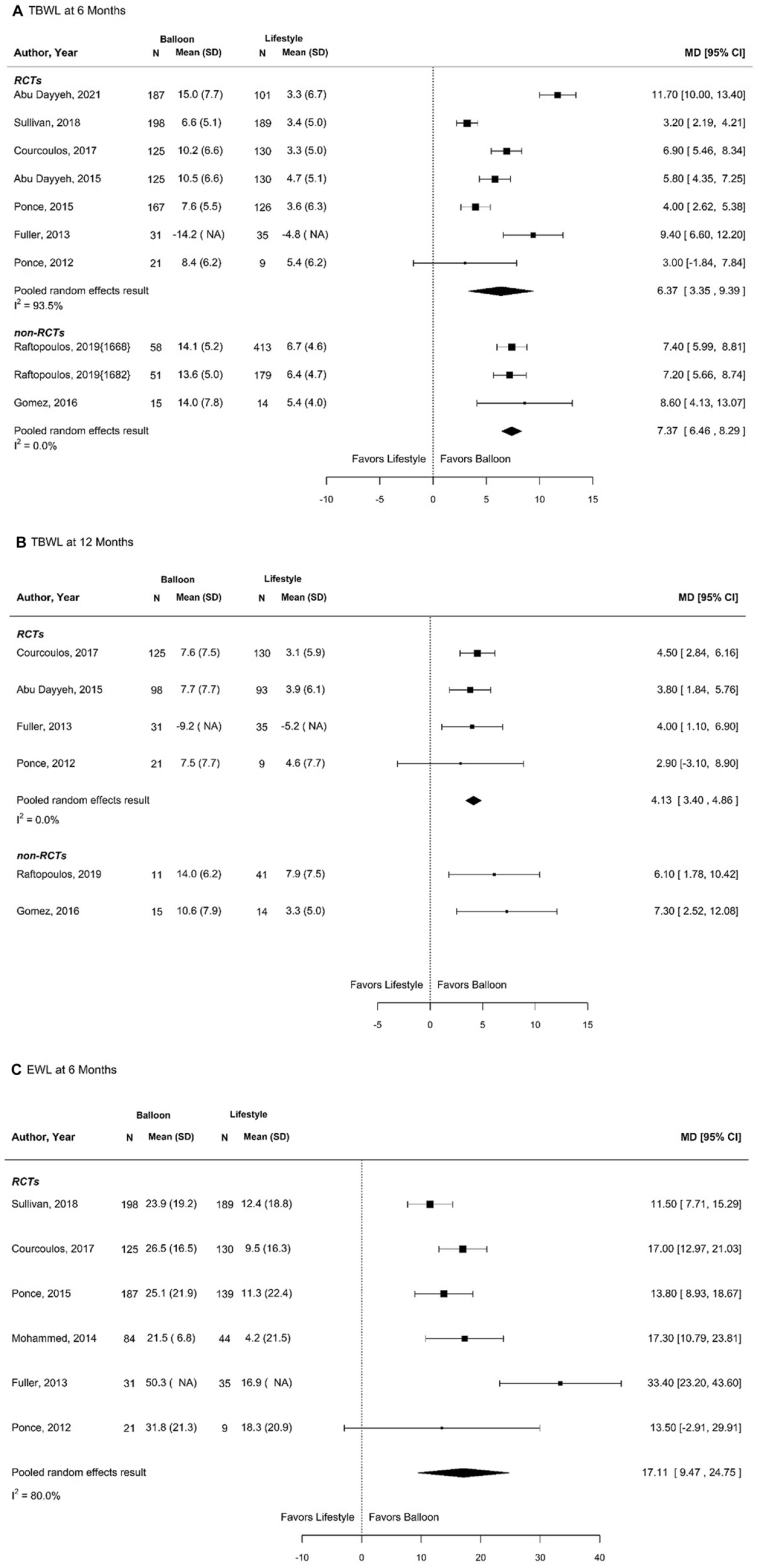
Meta-analyses of balloon versus lifestyle for varying outcomes

Figure 2A–C shows the results of the meta-analysis. The random effects pooled estimate from 7 RCTs reporting 6 month %TBWL (Fig. 2A) was a mean difference of 6.4% more weight loss in the IGB group (95% CI 3.9%–8.8%). There was substantial heterogeneity (*I*^2^ = 93.5%). There was no evidence of publication bias (Egger’s test *p* = 0.903, Begg’s test *p* = 0.239). Results from 3 observational studies were consistent with these pooled RCT results. The random effects pooled estimate from 4 RCTs reporting 12 month %TBWL (Fig. 2B) was a mean difference of 4.1% more weight loss in the IGB group (95% CI 3.0%–5.3%) (Fig. 2B) [7, 8, 14, 15]. There was no heterogeneity or evidence of publication bias (*I*^2^ = 0.00%, Egger’s test *p* = 0.198, Begg’s test *p* = 0.469). Results from 2 observational studies were consistent with these pooled RCT results. The random effects pooled estimate from 6 RCTs reporting 6 month %EBWL (Fig. 2C) was a mean difference of 17.1% more weight loss in the IGB group (95% CI 11.6%–22.6%). There was substantial heterogeneity (*I*^2^ = 80%), but no evidence of publication bias (Egger’s test *p* = 0.618, Begg’s test *p* = 0.333). There were no observational studies for comparison. There were too few studies reporting 12 month %EBWL to support a meta-analysis.

#### Adverse events

Two case series reported total complications. Major complications from these case series were 0.7% and 0.8% [35, 36]. All comparative studies reported significantly higher total complication rates with IGB compared to lifestyle therapy [risk differences ranging from 0.03–0.57, 95% CI 0, 0.64]. In addition, reinterventions were significantly higher after IGB compared to lifestyle in the only study that reported this outcome by Courcoulas et al. (7.5% vs 0%) [8]. The incidence of abdominal pain with IGB was reported at 5.29% in one case series [35]. Rates of nausea and vomiting were noted to be 63% and 31%, respectively, after IGB placement [35]. The incidence of gastric ulceration with IGB were reported as 0.82% and 0.9% in the two case series [35, 36]. Bleeding rates after IGB placement were reported as 0.01% and 0.6% [36, 37], and GERD was reported in 0.82% of cases [35]. Sullivan et al. compared bleeding rates between IGB and lifestyle therapy, showing no significant difference [10]. GERD rates were noted to be significantly higher after IGB compared to lifestyle by Courcoulas et al [8]. Adverse events data can be seen in Table 1.

### Endoscopic gastric plications (ESG and POSE)

#### Weight loss

**Fig. 3:**
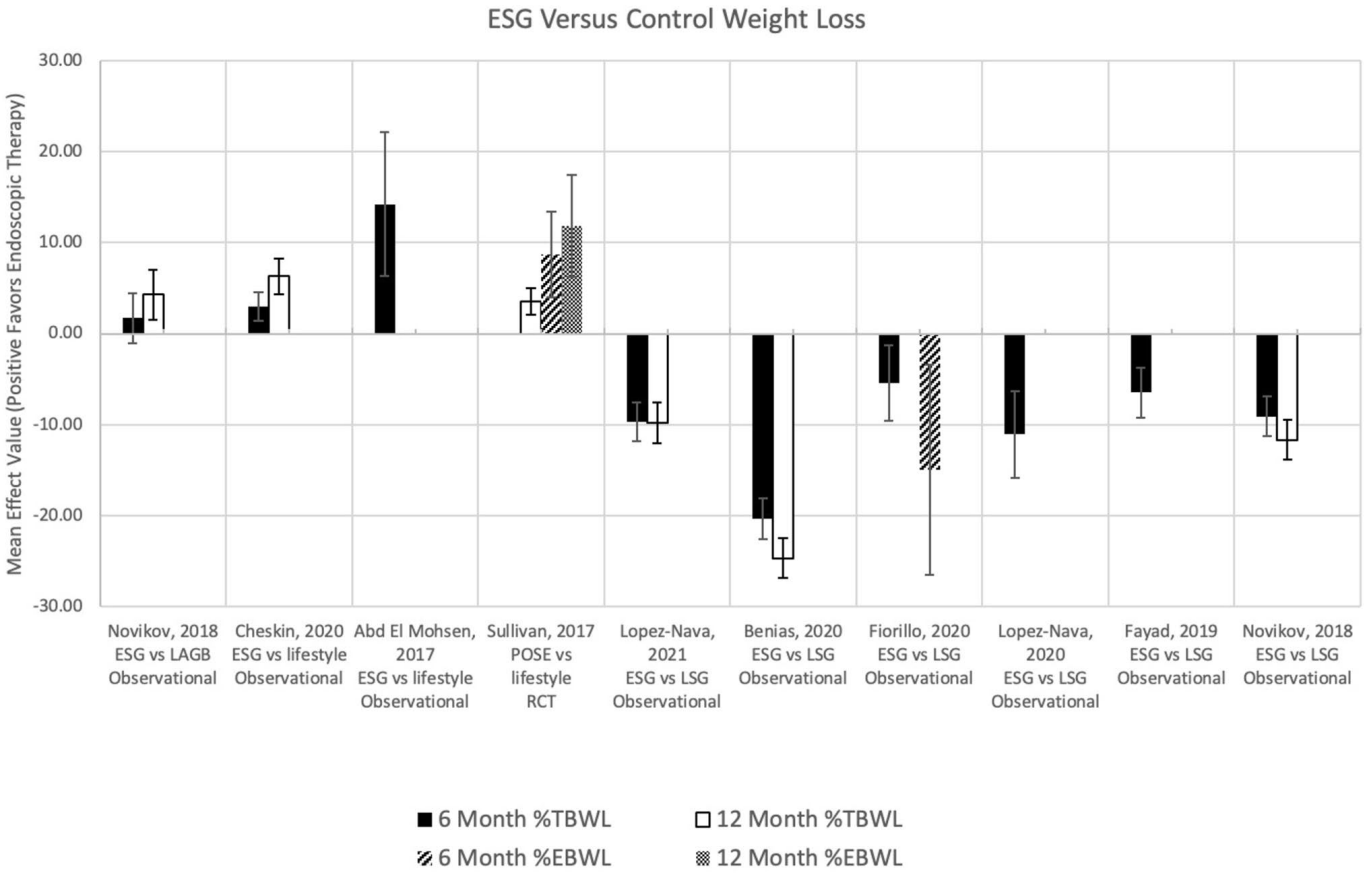
Weight loss outcomes for ESG versus other therapies at 6 and 12 months

Nine studies were identified, 2 of which comparing ESG to lifestyle modification, 1 comparing POSE to lifestyle modification, 1 comparing ESG to LAGB and LSG, and 5 comparing ESG to LSG (Fig. 3). At 6 months, 2 studies compared %TBWL after ESG or POSE versus lifestyle modification [23, 24]. All studies reported greater total body weight loss with both gastric plication techniques as compared to lifestyle, with mean weight loss differences of 3.0 and 14.5% TBWL for ESG and POSE, respectively.

Six studies compared %TBWL after ESG versus LSG at 6 months and found greater weight loss with LSG compared to ESG, with mean %TBWL after ESG between 4.7–14.4% compared to 18.8–26.5% after LSG and mean differences between 5.4–20.3% TBWL [25–30]. At 12 months, 3 studies reported %TBWL between 4.5–18.6% after ESG compared to 28.4–29.3% after LSG, with mean differences between 9.8–24.7% [25, 26, 30].

Excess body weight loss was evaluated in 2 studies. There was one RCT comparing POSE to lifestyle modification demonstrating greater weight loss with gastric plication (mean difference 8.7%EBWL at 6 months and 11.8%EBWL at 12 months) [24]. The other study, an observational study comparing ESG to LSG, showed significantly less weight loss with ESG (39.9% EBWL in ESG vs 54.9% EBWL in LSG patients, mean difference 15.0%) [27].

#### Adverse events

Four studies compared total complication rates between ESG and LSG, of which 3 reported no significant difference and one noted higher rates following LSG (Lopez-Nava 0.04 [− 0.01, 0.1]) [25–27, 29]. In the 3 studies evaluating readmission after ESG and LSG, there were reported no differences. at 30 days post-procedure [25, 27, 29]. Similarly, in the 3 studies evaluating bleeding, there was no difference reported with an incidence of 0.7% [26, 27, 29, 38]. Two studies compared rates of vomiting after ESG compared to LSG, Novikov et al. finding no difference and Fayad et al. reporting higher rates of vomiting after ESG compared to LSG [0.01 (0.01, 0.16)] [25, 29]. Finally, 2 studies evaluated GERD in these cohorts finding a significantly higher incidence of GERD after ESG compared to LSG (risk differences of 0.13 and − 0.32) [27, 29].

Compared to lifestyle modification, in the 2 studies that evaluated complications, total complications were higher after endoscopic plications (risk difference of 0.09 [0.01, 0.17] [24] and 0.05 [0.01, 0.09] [22]), nausea and vomiting were more likely [risk differences of 0.14 (0.07, 0.21) and 0.19 (0.13, 0.24), respectively] [24] and GERD was no different [0.04 (− 0.02, 0.09)] [24].

### AspireAssist

#### Weight loss

Three studies were identified, 2 comparing AspireAssist to lifestyle modification [32, 33] and one to RYGB [34]. Both RCTs comparing AspireAssist to lifestyle modification demonstrated statistically significant increased %TBWL with aspiration therapy compared to lifestyle modification at 12 months, 12.1% and 18.3% TBWL versus 3.5% and 5.9% TBWL with mean differences of 8.8% and 12.4% [32, 33]. Aspiration therapy was associated with less %TBWL at 12 months compared to RYGB, 20.0% versus 32.0%TBWL with mean difference of 12.0% [34].

Two RCTs compared %EBWL after AspireAssist as compared to lifestyle modification. One study reported data for each arm after 12 months, with 49.0%EBWL after aspiration therapy vs 14.9%EBWL with lifestyle modification, mean difference 34.1% [33]. One observational study reported significantly greater %EBWL at 12 months with RYGB compared to AspireAssist (85.0% EBWL with RYGB compared to 52.0%EBWL with aspiration therapy, mean difference 33.0%) [34].

#### Adverse events

No large volume case series reported total complication rates after aspiration therapy. The single comparative study reporting by Thompson et al. found higher rates of total complications after AspireAssist compared to lifestyle therapy, risk difference 0.78 (0.71, 0.86). No data was available regarding readmissions or reintervention rates compared to lifestyle therapy [32]. However, compared to lifestyle, Thompson et al. found abdominal pain and vomiting were more common after aspiration therapy, risk differences of 0.38 and 0.17, respectively.

### Secondary outcomes

#### Quality of life (QoL) and HgA1c

There were limited studies focusing on QoL outcomes (Table 2). For IGB, one study evaluated QoL at 6 and 12 months after IGB placement compared to lifestyle modification, finding mean differences in IWQOL-Lite scores 7.5 points and 6.4 points greater at 6 months and 12 months, respectively, after IGB compared to lifestyle, though significance could not be determined [8]. Two studies reported QoL outcomes after ESG compared to lifestyle modification. One study found mean difference in IWQOL-Lite scores 3.1 points greater after ESG compared to lifestyle modification at 6 months [21]. Two studies evaluated changes in QoL after ESG compared to LSG of which one found no significant differences in GI-QOL scores, while the second reported 7.9% less improvement in QoL after ESG compared to LSG on a subjective scale of 1–10 at 6 months [27, 31].

In regards to HgA1C, one study reported changes in HbA1c after IGB compared to lifestyle modification, finding no significant difference between the groups (mean difference 0.0, CI − 0.14–0.14) [10]. Another study found no statistically different change in HbA1c after ESG compared to lifestyle modification (mean differences − 0.03 for both arms) [13].

### Certainty of evidence

To make determinations about certainty of evidence, we factored in assessments of risk of bias when judging the degree of study limitations and considered all studies to satisfy the directness domain, as all studies measured weight loss, quality of life, a metabolic outcome, or complications in standard ways. We drew on the large body of literature about lifestyle therapy for weight loss to conclude that complications like reintervention, bleeding, and ulceration can safely be assumed to be negligible with lifestyle therapy. Of the conclusions drawn, we judged that the following conclusions have high certainty of evidence: IGB achieves greater %TBWL than lifestyle therapy at 6 and 12 months, IGB achieves more %EBWL than lifestyle therapy at 6 months, ESG achieves more %TBWL than lifestyle therapy at 6 months, and AspireAssist, IGB, and ESG each have greater total complications than lifestyle therapy. A complete description of certainty of evidence can be found in Online Appendix F.

## Discussion

This review found that regulator approved EBTs (intragastric balloons, ESG/POSE, and AspireAssist) are associated with greater weight loss at 6 or 12 months when compared to lifestyle modification alone. These findings were consistent across RCTs and observational studies. This review also found that treatment with ESG is associated with less weight loss than LSG, although this conclusion is based solely on observational studies. Similarly, AspireAssist was associated with less weight loss than RYGB but quality of data is low given results are drawn from a single observational study.

A joint taskforce compromising of the American Society of Gastrointestinal Endoscopy (ASGE) and the American Society of Metabolic and Bariatric Surgery (ASBMS) defined certain therapeutic thresholds to consider for adoption of endoscopic bariatric therapies [39]. They recommended a minimum of 25% EBWL at 12 months for primary therapies and 5% TWL overall for bridging therapies. The outcomes for IGBs, ESG and AspireAssist all surpass these outcome measures and meet criteria for incorporation for both primary and bridging therapies within clinical practice. The ASGE recommendsthat the incidence of serious adverse events related to EBTs should be < 5% for consideration of adoption into clinical practice [39]. All studied endoscopic bariatric therapies safety profiles were acceptable based on these standards, and most complications were managed conservatively without endoscopic or surgical re-intervention. As expected, studies comparing endoscopic bariatric therapy to lifestyle reported more total complications and 30-day readmission or re-intervention rates in the intervention arm, given these patients underwent an invasive procedure. There were no or borderline statistically significant differences in total complications between patients treated with LSG compared to ESG, although all studies reported more complications with LSG.

Our study has several limitations. First, there were no randomized trials or observational studies directly comparing endoscopic bariatric therapies to existing pharmacotherapy that met inclusion criteria for this analysis. Recent short and long term data from the STEP 5 trial showed the value of pharmacologic therapy (e.g., semaglutide) compared to lifestyle modification in weight loss management for obesity; however, no such trial has yet been performed comparing pharmacologic therapy to endoscopic or surgical therapy. This topic deserves further high-quality studies when determining the spectrum of management for patients with obesity [40]. Second, long term data on endoscopic interventions was limited in these studies with many fewer studies reporting 12 month outcomes than 6 month outcomes. This limits the conclusions that can be stated regarding recidivism and durability. Third, potential selection bias exists in these studies for multiple reasons. To more comprehensively assess the topic, we included observational studies, especially for ascertaining adverse event profiles for the various interventions. Many observational studies did not discuss how patients were directed to either EBT or control, causing potential for selection bias. Also, while multiple RCTS were included and were judged to have low risk of bias, true blinding of participants is difficult with bariatric interventions, and even with high-quality sham controlled swallowable balloon protocols, it is likely patients were able to detect which arm they were assigned. Fourth, there were no RCTs comparing endoscopic technique to endoscopic technique such that no comment can be made on the value of one compared to another. A corollary to this is the heterogeneity of specific devices. Whether swallowed or endoscopically placed, these devices have differing characteristics which may play a role in clinical outcomes and were unable to be assessed.

An additional limitation is heterogeneity in the results for weight loss at 6 months comparing balloon treatment with lifestyle management. This heterogeneity is caused by 3–fourfold differences in the point estimate of effect between studies by Abu Dayyeh and Fuller with the study by Sullivan. This heterogeneity may be attributed to balloon type, as Abu Dayyeh and Fuller reported outcomes after Orbera balloon placement while Sullivan studied the Elipse balloon, though this is speculation given available data. Interestingly, at 12 months there is no heterogeneity in estimates of weight loss outcomes, possibly because the study by Sullivan did not report 12-month outcomes [7, 10, 15]. Additional study heterogeneity exists for duration of balloon placement, since some studies replaced balloons at 6 months for a total duration of 12 months, while others only left balloons in place for 12 months.

The field of bariatric and metabolic medicine is diverse, consisting of lifestyle, pharmacologic, surgical, and now endoscopic therapies. The most widely adopted endoscopic therapy is intragastric balloons due to the technical ease of insertion and removal. Endoscopic plications (ESG and POSE) are effective but technically challenging and offered only at specialized centers. We note that during our search query Aspire Assist was available in the market for clinical use. However, it was recently removed from the market by Aspire Bariatrics as of April 2022, though not because of clinical concerns or adverse events. While bariatric surgery is the gold standard treatment for obesity with a high degree of evidence suggesting that LSG and RYGB result in greater weight loss than endoscopic or pharmacologic therapies, our data suggest a distinct role for endoscopic therapies. Obesity remains highly prevalent in the United States with a comparatively low utilization of bariatric surgery, such that there exist opportunities for additional treatments for obesity, like endoscopic therapies and pharmacologic therapies. Our study emphasizes the need to tailor bariatric interventions for specific patient needs. In terms of long term weight loss, bariatric surgery remains the standard therapy, as this study found it remains to be demonstrated how durable endoscopic bariatric therapies like IGB and ESG remain over 12 months post-procedure. However, in patients who may not have access to bariatric surgery or may not be candidates, our study demonstrates with high certainty of evidence that endoscopic therapies yield significantly greater weight loss than lifestyle therapy alone. This may be useful in patients who need temporary weight loss to allow candidacy for other procedures, such as hernia repair, in which high BMI patients have higher rates of treatment failure and complications, or other patients who would benefit from significant short term weight loss. Additionally, our study demonstrates that endoscopic therapies achieve no significant difference in HgbA1c reduction compared to lifestyle therapy. Though not specifically studied in diabetics, further studies may be necessary to investigate whether patients with diabetes should preferentially undergo other bariatric treatments, such as surgery or GLP-1 agonist pharmacotherapy.

## Conclusions

In general, the data suggests that endoscopic bariatric therapies play a role in weight loss management, as they are more effective than lifestyle modification yet less effective than surgery. Nevertheless, more robust data in the form of RCTs or case-controlled studies are needed. Our meta-analyses demonstrate a possible middle ground for the consideration of endoscopic bariatric therapies for patients who require significantly more weight loss than lifestyle therapy, but may not qualify or wish for surgical intervention despite the potential for significantly more weight loss. Although limited by high-quality randomized data, there appears to be a role for endoscopic bariatric therapies in the management spectrum for patients with obesity.

### Supplementary Information

Below is the link to the electronic supplementary material.

Supplementary file1 (DOCX 14 KB) Search strategy

Supplementary file2 (DOCX 160 KB) Literature flow

Supplementary file3 (DOCX 15 KB) Quality assessment for included RCT studies

Supplementary file4 (DOCX 15 KB) Quality assessment for included observational studies

Supplementary file5 (DOCX 16 KB) Table of frequencies of included outcomes

Supplementary file6 (DOCX 17 KB) Grade certainty of evidence

Supplementary file7 (DOCX 20 KB) Evidence table

## Data Availability

All data collected for this research are reported in the manuscript and accompanying supplement and will be made available to other researchers.
