# Supplementary material for: Endoscopic therapies for patients with obesity: a systematic review and meta-analysis
Source: Surg Endosc. 2023 Sep 20;37(11):8166–77. doi: 10.1007/s00464-023-10390-6 (PMC10615978; doi:10.1007/s00464-023-10390-6)
Supplement: Supplementary file 1 — Supplementary file1 (DOCX 14 KB) Search strategy [file 464_2023_10390_MOESM1_ESM.docx]

Appendix A

**Appendix A: Search Strategy**

**PubMed**

((("Aspiration therapy"[Title/Abstract] OR AspireAssist[Title/Abstract] OR "Intragastric balloon"[Title/Abstract] OR "endoscopic sleeve gastroplasty"[Title/Abstract] OR "endoscopic sleeve"[Title/Abstract] OR "Endoscopic gastroplasty"[Title/Abstract] OR "primary obesity surgery endoluminal"[Title/Abstract] OR "Endoscopic gastric plication"[Title/Abstract] OR "gastric volume reduction"[Title/Abstract] OR OverStitch[Title/Abstract] OR ORBERA[Title/Abstract] OR Obalon[Title/Abstract] OR "Gastric Balloon"[Title/Abstract] OR Gastrostomy[Title/Abstract] OR Gastroplasty[Title/Abstract] OR "Collis Gastroplasty"[Title/Abstract] OR "Vertical Banded Gastroplasty"[Title/Abstract] OR "Gastrostomies"[Title/Abstract] OR "Gastric Bubble"[Title/Abstract] OR IGB[Title/Abstract] OR ESG [Title/Abstract])) OR ((Gastric Balloon [MeSH] OR Gastrostomy [MeSH] OR Gastroplasty [MeSH]))) AND ((("metabolic Surgery"[Title/Abstract] OR "Bariatric Surgery"[Title/Abstract] OR "Bariatric Surgical Procedure"[Title/Abstract] OR "Stomach Stapling"[Title/Abstract] OR "gastric bypass"[Title/Abstract] OR MGB[Title/Abstract] OR OAGB[Title/Abstract] OR lifestyle[Title/Abstract] OR "life style"[Title/Abstract] OR lifestyles[Title/Abstract] OR "life styles"[Title/Abstract] OR “Lifestyle therapy”[Title/Abstract])) OR (("Bariatric Surgery"[Mesh] OR "gastric bypass"[Mesh] OR Life Style[MESH])))

Filters: from 2014/1/1 - 2021/12/07 [1599]

AND

“endoscopic bariatric therapy”[Title/Abstract]

Filters: from 2014/1/1 – 2022/01/23 [45]

**Embase**

('aspiration therapy':ab,ti OR aspireassist:ab,ti OR 'intragastric balloon':ab,ti OR 'endoscopic sleeve gastroplasty':ab,ti OR 'endoscopic sleeve':ab,ti OR 'endoscopic gastroplasty':ab,ti OR 'primary obesity surgery endoluminal':ab,ti OR 'endoscopic gastric plication':ab,ti OR 'gastric volume reduction':ab,ti OR overstitch:ab,ti OR orbera:ab,ti OR obalon:ab,ti OR 'gastric balloon':ab,ti OR gastrostomy:ab,ti OR gastroplasty:ab,ti OR 'collis gastroplasty':ab,ti OR 'vertical banded gastroplasty':ab,ti OR 'gastrostomies':ab,ti OR 'gastric bubble':ab,ti OR igb:ab,ti OR esg:ab,ti OR (“gastric balloon”/exp) OR 'gastrostomy'/exp OR 'gastroplasty'/exp) AND ('metabolic surgery':ab,ti OR 'bariatric surgery':ab,ti OR 'bariatric surgical procedure':ab,ti OR 'stomach stapling':ab,ti OR 'gastric bypass':ab,ti OR mgb:ab,ti OR oagb:ab,ti OR lifestyle:ab,ti OR 'life style':ab,ti OR lifestyles:ab,ti OR 'life styles':ab,ti OR 'lifestyle therapy':ab,ti OR ‘endoscopic bariatric therapy’:ab,ti OR (('bariatric surgery'/exp OR 'gastric bypass'/exp OR “life style”/exp))

Filters: from 2014/1/1 - 2021/12/07 [1533]

AND

‘endoscopic bariatric therapy’:ab,ti

Filters: from 2014/1/1 – 2022/01/23 [36]

**Cochrane**

("Aspiration therapy" OR AspireAssist OR "Intragastric balloon" OR "endoscopic sleeve gastroplasty" OR "endoscopic sleeve" OR "Endoscopic gastroplasty" OR "primary obesity surgery endoluminal" OR "Endoscopic gastric plication" OR "gastric volume reduction" OR OverStitch OR ORBERA OR Obalon OR "Gastric Balloon" OR Gastrostomy OR Gastroplasty OR "Collis Gastroplasty" OR "Vertical Banded Gastroplasty" OR "Gastrostomies" OR "Gastric Bubble" OR IGB OR ESG) in Title Abstract Keyword OR (mh Gastroplasty OR mh Gastrostomy OR mh "Gastric Balloon") in All Text OR (mh "Bariatric Surgery" OR mh "gastric bypass" OR mh "Life Style") in All Text AND ("metabolic Surgery" OR "Bariatric Surgery" OR "Bariatric Surgical Procedure" OR "Stomach Stapling" OR "gastric bypass" OR MGB OR OAGB OR lifestyle OR "life style" OR lifestyles OR "life styles" OR “Lifestyle therapy”) in Title Abstract Keyword - with Cochrane Library publication date Between Jan 2014 and Dec 1 [985]

AND

“endoscopic bariatric therapy”

Between Jan 2014 and Jan 2022 [5]
