# Supplementary figures and images for: Endoscopic therapies for patients with obesity: a systematic review and meta-analysis
Source: Surg Endosc. 2023 Sep 20;37(11):8166–77. doi: 10.1007/s00464-023-10390-6 (PMC10615978; doi:10.1007/s00464-023-10390-6)

Appendix B


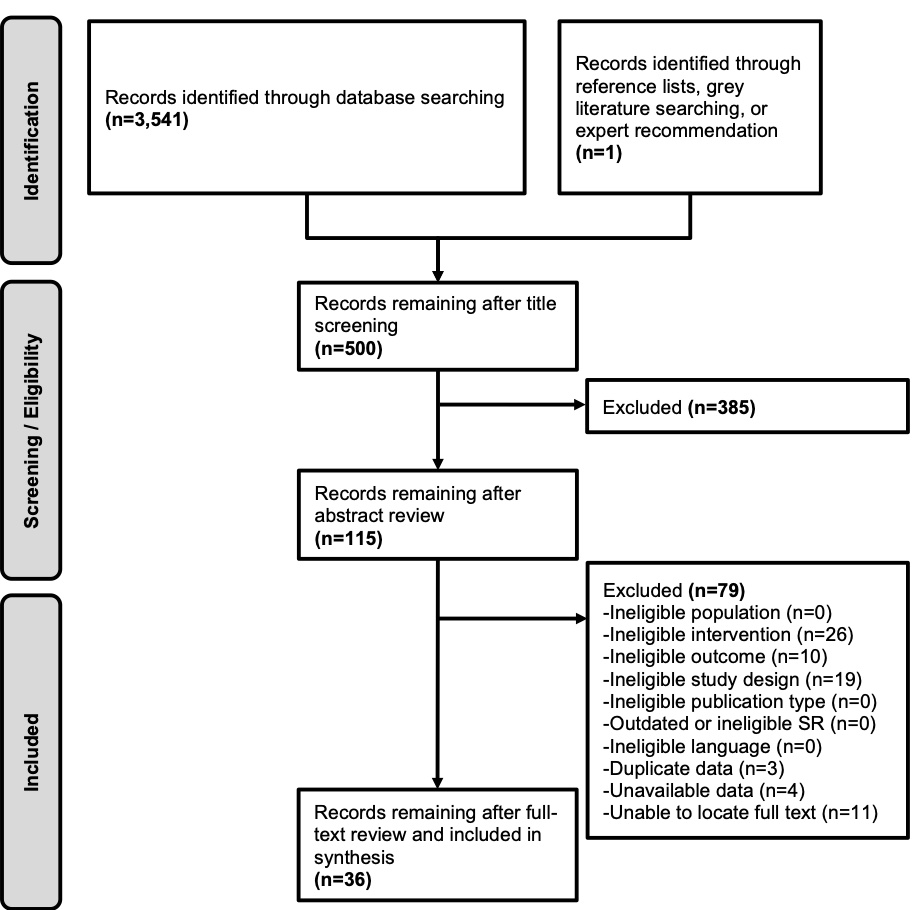

Supplement: Supplementary file 2 — Supplementary file2 (DOCX 160 KB) Literature flow [file 464_2023_10390_MOESM2_ESM.docx]
