# Supplementary material for: Endoscopic therapies for patients with obesity: a systematic review and meta-analysis
Source: Surg Endosc. 2023 Sep 20;37(11):8166–77. doi: 10.1007/s00464-023-10390-6 (PMC10615978; doi:10.1007/s00464-023-10390-6)
Supplement: Supplementary file 3 — Supplementary file3 (DOCX 15 KB) Quality assessment for included RCT studies [file 464_2023_10390_MOESM3_ESM.docx]

**Appendix C: Quality Assessment for Included RCT Studies**

| **Author, year** | **Random sequence generation** | **Allocation concealment** | **Blinding of participants and personnel** | **Blinding of outcome assessment** | **Incomplete outcome data at 6, 12 months, longest** | **Selective reporting** | **Other sources of bias** |
| --- | --- | --- | --- | --- | --- | --- | --- |
| Abu Dayyeh, 2015 | Unknown risk | Unknown risk | High risk | High risk | High risk (70-75% follow up at 1 year) | Low risk | Moderate risk |
| Abu Dayyeh, 2019 | Unknown risk | Unknown risk | High risk | High risk | Low risk | Low risk | Moderate risk |
| Chan, 2021 | Unknown risk | Unknown risk | High risk | High risk | Moderate risk | Low risk | Low risk |
| Courcoulas, 2017 | Low risk (Trial is FDA approved) | Unknown risk | High risk | Unknown risk for weight  Unclear how weight measured  Moderate risk for QOL | Low risk at 9 months (80% follow up)  High risk at 12 months (75% follow up) | Low risk | Moderate risk |
| Fuller, 2013 | Unknown risk | Unknown risk | High risk | High risk | Low risk | Low risk | Moderate risk |
| Gomez, 2016 | Low risk  (Trial is FDA approved) | Unknown risk | High risk | Unknown risk for weight  Unclear how weight measured  Moderate risk for QOL  High risk for gastric emptying | Low risk (100% follow up) | High risk Weight is not primary endpoint of study | Moderate risk |
| Lee, 2012 | Unknown risk | Unknown risk | Moderate risk | Moderate risk | Low risk | Low risk | Low risk |
| Mohammed, 3220 | Unknown risk | Unknown risk | High risk | Moderate risk | Low risk | Low risk | Low risk |
| Ponce, 2013 | Unknown risk | Unknown risk | Moderate risk | Moderate risk | Low risk | Low risk | Moderate risk |
| Ponce, 2015 | Low risk | Low risk | Moderate risk | Moderate risk | Low risk | Low risk | Moderate risk |
| Raftoupoulos, 2019 | Unknown risk | Unknown risk | High risk | High risk | High risk | Low risk | Low risk |
| Sullivan, 2012 | Unknown risk | Unknown risk | High risk | High risk | Low risk | Low risk | Low risk |
| Sullivan, 2017 | Low risk  (Trial is FDA approved) | Unknown risk | Low risk | Unknown risk for weight Unclear how weight measured Moderate risk for QOL | Low risk | Low risk | Moderate risk |
| Sullivan, 2018 | Low risk  (Trial is FDA approved) | Unknown risk | Low risk | Unknown risk for weight Unclear how weight measured Moderate risk for QOL | Los risk | Low risk | Moderate risk |
| Thompson, 2017 | Low risk  (Trial is FDA approved) | Unknown risk | High risk | Unknown risk for weight Unclear how weight measured Moderate risk for QOL | High risk | Low risk | Moderate risk |
| Thompson, 2019 | Low risk  (Trial is FDA approved) | Unknown risk | High risk | Unknown risk for weight Unclear how weight measured Moderate risk for QOL | High risk | Low risk | Moderate risk |
