# Supplementary material for: Endoscopic therapies for patients with obesity: a systematic review and meta-analysis
Source: Surg Endosc. 2023 Sep 20;37(11):8166–77. doi: 10.1007/s00464-023-10390-6 (PMC10615978; doi:10.1007/s00464-023-10390-6)
Supplement: Supplementary file 4 — Supplementary file4 (DOCX 15 KB) Quality assessment for included observational studies [file 464_2023_10390_MOESM4_ESM.docx]

Appendix D

| **Author, year** | **Bias due to confounding** | **Bias in selection of participants** | **Bias in classification of interventions** | **Bias due to deviations from intended interventions** | **Bias due to missing data** | **Bias in measurement of outcomes** | **Bias in selection of reported results** |
| --- | --- | --- | --- | --- | --- | --- | --- |
| Abd El Mohsen, 2017 | Unknown risk | Unknown risk | Low risk | Low risk | Low risk | Moderate risk | Low risk |
| Abeid, 2019 | Low risk | Low risk | Low risk | Low risk | Low risk | Moderate risk | Low risk |
| Ahmed, 2019 | Moderate risk | Moderate risk | Low risk | Low risk | Low risk | High risk | Low risk |
| Alqahtani, 2019 | Low risk | Low risk | Low risk | Low risk | Low risk | Moderate risk | Low risk |
| Benias, 2020 | Low risk | Moderate risk | Low risk | Low risk | Unknown risk | Moderate risk | Low risk |
| Cheskin, 2020 | Low risk | Low risk | Low risk | Low risk | High risk | Moderate risk | Low risk |
| Espinet Coll, 2017 | Low risk | Low risk | Low risk | Low risk | Low risk | Moderate risk | Low risk |
| Fayad, 2019 | Moderate risk | Low risk | Low risk | Low risk | High risk | Moderate risk | Low risk |
| Fiorillo, 2020 | Low risk | High risk | Low risk | Low risk | Low risk | Moderate risk | Low risk |
| Lopez-Nava, 2020 | Low risk | Unknown risk | Low risk | Low risk | Low risk | Low risk | Low risk |
| Lopez-Nava, 2021 | Low risk | High risk | Low risk | Low risk | Low risk | Moderate risk | Low risk |
| Mathus-Vliegen, 2015 | Low risk | Moderate risk | Low risk | Low risk | Low risk | Moderate risk | Low risk |
| Novikov, 2018 | High risk | High risk | Low risk | Low risk | Low risk | Moderate risk | Low risk |
| Raftopoulos, 2019 | Moderate risk | Unknown risk | Low risk | Low risk | Unknown risk | Moderate risk | Low risk |
| Raftopoulos, 2019 | Unknown risk | Unknown risk | Low risk | Low risk | Unknown risk | Moderate risk | Unknown risk |
| Sadek, 2017 | Unknown risk | Unknown risk | Low risk | Low risk | Unknown risk | Moderate risk | Low risk |
| Salomone, 2021 | Moderate risk | Low risk | Low risk | Low risk | Low risk | Moderate risk | Low risk |
| Sander, 2017 | Low risk | Moderate risk | Low risk | Low risk | Low risk | Moderate risk | Low risk |
| Wilson, 2018 | Low risk | Moderate risk | Low risk | Low risk | Low risk | Moderate risk | Low risk |
