# Supplementary material for: Endoscopic therapies for patients with obesity: a systematic review and meta-analysis
Source: Surg Endosc. 2023 Sep 20;37(11):8166–77. doi: 10.1007/s00464-023-10390-6 (PMC10615978; doi:10.1007/s00464-023-10390-6)
Supplement: Supplementary file 5 — Supplementary file5 (DOCX 16 KB) Table of frequencies of included outcomes [file 464_2023_10390_MOESM5_ESM.docx]

Appendix E

| **Outcome** | **f/u Time (months)** | **# Studies** | **# RCTs** | **# nonRCTs** |
| --- | --- | --- | --- | --- |
| %TBWL | 6 | 19 | 7 | 12 |
| %TBWL | 12 | 15 | 7 | 8 |
| %EBWL | 6 | 8 | 7 | 1 |
| %EBWL | 12 | 8 | 7 | 1 |
| BMI | 6 | 8 | 6 | 2 |
| total complication | 6 | 7 | 4 | 3 |
| weight loss | 6 | 7 | 6 | 1 |
| weight loss | 12 | 7 | 6 | 1 |
| nausea/vomiting | 12 | 6 | 3 | 3 |
| total complication | 12 | 6 | 3 | 3 |
| BMI | 12 | 5 | 3 | 2 |
| QOL | 6 | 5 | 3 | 2 |
| infection | 12 | 5 | 2 | 3 |
| GERD | 6 | 4 | 2 | 2 |
| at least 10% total body weight | 6 | 4 | 3 | 1 |
| at least 5% total body weight | 6 | 4 | 3 | 1 |
| bleeding | 12 | 4 | 2 | 2 |
| dehydration | 6 | 4 | 3 | 1 |
| nausea/vomiting | 6 | 4 | 3 | 1 |
| %TBWL | 24 | 3 | 0 | 3 |
| 30-day readmissions (time not specified) | 6 | 3 | 1 | 2 |
| 30-day reintervention | 12 | 3 | 1 | 2 |
| QOL | 12 | 3 | 3 | 0 |
| at least 10% total body weight | 12 | 3 | 3 | 0 |
| at least 25% excess body weight | 12 | 3 | 3 | 0 |
| at least 5% total body weight | 12 | 3 | 3 | 0 |
| bleeding | 6 | 3 | 1 | 2 |
| dehydration | 12 | 3 | 1 | 2 |
| dyspepsia/abdominal pain | 6 | 3 | 3 | 0 |
| dyspepsia/abdominal pain | 12 | 3 | 2 | 1 |
| gastric ulceration | 6 | 3 | 3 | 0 |
| HbA1C | 6 | 3 | 3 | 0 |
| HbA1C | 12 | 3 | 2 | 1 |
| 30-day reintervention | 6 | 2 | 1 | 1 |
| BMI | 24 | 2 | 0 | 2 |
| GERD | 12 | 2 | 2 | 0 |
| at least 25% excess body weight | 6 | 2 | 2 | 0 |
| infection | 6 | 2 | 1 | 1 |
| mortality | 12 | 2 | 1 | 1 |
| weight loss | 24 | 2 | 1 | 1 |
| %EBWL | 24 | 1 | 0 | 1 |
| %EBWL | 36 | 1 | 0 | 1 |
| %TBWL | 36 | 1 | 0 | 1 |
| %TBWL | 120 | 1 | 1 | 0 |
| 30-day readmissions (only know within 90 days though) | 12 | 1 | 0 | 1 |
| 30-day readmissions (time not specified) | 12 | 1 | 1 | 0 |
| 30-day reintervention (don't know time frame) | 12 | 1 | 0 | 1 |
| BMI | 120 | 1 | 1 | 0 |
| gastric ulceration | 12 | 1 | 0 | 1 |
| improvement of diabetes | 12 | 1 | 1 | 0 |
| improvement of hypertension | 12 | 1 | 1 | 0 |
| weight loss | 60 | 1 | 1 | 0 |
| weight loss | 120 | 1 | 1 | 0 |
