# Supplementary material for: Endoscopic therapies for patients with obesity: a systematic review and meta-analysis
Source: Surg Endosc. 2023 Sep 20;37(11):8166–77. doi: 10.1007/s00464-023-10390-6 (PMC10615978; doi:10.1007/s00464-023-10390-6)
Supplement: Supplementary file 6 — Supplementary file6 (DOCX 17 KB) Grade certainty of evidence [file 464_2023_10390_MOESM6_ESM.docx]

Appendix F

| **Outcome** | **Study Limitations** | **Consistency** | **Directness** | **Precision** | **Certainty of Evidence** |  |
| --- | --- | --- | --- | --- | --- | --- |
| **%TBWL** |  |  |  |  |  |  |
| *At 6 months* |  |  |  |  |  |  |
| IBG > Lifestyle | RCT: No serious limitations  Unmatched observational studies: Serious | Consistent | Direct | Precise | High |  |
| ESG > Lifestyle | Matched observational studies: Serious  Unmatched observational studies: Serious | Consistent | Direct | Imprecise | Low |  |
| ESG < LSG | Matched observational studies: Serious  Unmatched observational studies: Serious | Consistent | Direct | Precise | Low |  |
| *At 12 months* |  |  |  |  |  |  |
| AspireAssist > Lifestyle | RCT: No serious limitations | N/A | Direct | Precise | Moderate |  |
| AspireAssist < RYGB | Unmatched observational studies: Serious limitations | N/A | Direct | Precise | Low |  |
| IGB > Lifestyle | RCT: No serious limitations  Unmatched observational studies: Serious limitations | Consistent | Direct | Precise | High |  |
| ESG > Lifestyle | RCT: No serious limitations | Consistent | Direct | Precise | High |  |
| ESG < LSG | Unmatched observational studies: Serious limitations | Consistent | Direct | Precise | Low |  |
| **%EBWL** |  |  |  |  |  |  |
| *At 6 months* |  |  |  |  |  |  |
| IGB > Lifestyle | RCT: No serious limitations | Consistent | Direct | Precise | High |  |
| ESG > Lifestyle | RCT: No serious limitations | N/A | Direct | Precise | Moderate |  |
| ESG < LSG | Matched observational studies: Serious limitations | N/A | Direct | Precise | Low |  |
| *At 12 months* |  |  |  |  |  |  |
| AspireAssist > Lifestyle | RCT: No serious limitations | N/A | Direct | Precise | Moderate |  |
| AspireAssist < RYGB | Unmatched observational studies: Serious limitations | N/A | Direct | Precise | Low |  |
| IGB > Lifestyle | RCT: No serious limitations | N/A | Direct | Precise | Moderate |  |
| ESG > Lifestyle | RCT: No serious limitations | N/A | Direct | Precise | Moderate |  |
| **HbA1C** |  |  |  |  |  |  |
| *At 6 months* |  |  |  |  |  |  |
| IGB = Lifestyle | RCT: No serious limitations | N/A | Direct | Precise | Moderate |  |
| ESG = Lifestyle | RCT: No serious limitations | N/A | Direct | Precise | Moderate |  |
| AspireAssist = Lifestyle | RCT: Very serious limitations | N/A | Direct | N/A | Very low |  |
| **Quality of Life** |  |  |  |  |  |  |
| *At 12 months* |  |  |  |  |  |  |
| ESG > Lifestyle | RCT: No serious limitations | N/A | Direct | Precise | Low |  |
| ESG = LSG | Unmatched observational studies: Serious limitations | N/A | Direct | Imprecise | Very Low |  |
| **Total Complications** | | | | | | |
| *At 6 months* |  |  |  |  |  |  |
| AspireAssist > Lifestyle | RCT: No serious limitations | N/A | Direct | Precise | High |  |
| IGB > Lifestyle | RCT: No serious limitations | Consistent | Direct | Precise | High |  |
| ESG > Lifestyle | RCT: No serious limitations | Consistent | Direct | Precise | High |  |
| ESG = LSG | Matched observational studies: Serious limitations  Unmatched observational studies: Serious limitations | Consistent | Direct | Precise | Low |  |
| *Abbreviations.* ESG=endoscopic sleeve gastroplasty; IGB=intragastric balloon; LSG=laparoscopic sleeve gastrectomy; RYGB=Roux-en-Y gastric bypass. | | | | | | |
