# Supplementary material for: Endoscopic therapies for patients with obesity: a systematic review and meta-analysis
Source: Surg Endosc. 2023 Sep 20;37(11):8166–77. doi: 10.1007/s00464-023-10390-6 (PMC10615978; doi:10.1007/s00464-023-10390-6)
Supplement: Supplementary file 7 — Supplementary file7 (DOCX 20 KB) Evidence table [file 464_2023_10390_MOESM7_ESM.docx]

Appendix G

|  | **Study Design / US** | **Single vs Multi- center** | **Propensity Matching** | **Patient Characteristics Preop** | | | | | |
| --- | --- | --- | --- | --- | --- | --- | --- | --- | --- |
|  |  |  |  | ESG | Balloon | AspireAssist | RYGB | LSG | Lifestyle |
| Cheskin, 2020 | Obs / Y | Single | Yes | n: 105 Age: 47.58 (11.97) Female: 75 (71.42) BMI: 40.50 (7.89) |  |  |  |  | n: 281 Age: 48.17 (12.18) Female: 189 (67.26) BMI: 39.85 (7.62) |
| Courcoulas, 2017 | RCT / Y | Multi | No |  | n: 125 Age: 38.7 (9.37) Race White: 101 (80.8) Black: 13 (11.2) Asian: 0 Hispanic: 9 (7.2) Female: 112 (89.6) BMI:  <30: 2 (1.6)  30-35: 63 (50.4)  35-40: 56 (44.8)  >40: 4 (3.2) EBW: 36# (11) DM: 9 (7) HTN: 33 (26) |  |  |  | n: 130 Age: 40.8 (9.61) Race White: 106 (81.5) Black: 15 (11.5) Asian: 0 Hispanic: 7 (5.4) Female: 117 (90.0) BMI:  <30: 1 (0.8)  30-35: 57 (43.8)  35-40: 70 (53.8)  >40: 2 (1.5) EBW: 36# (9) DM: 8 (6) HTN: 37 (28) |
| Fayad, 2019 | Obs / Y | Single | Yes | n: 54 Age: 48 (24-72) Female: 57.4% BMI: median BMI 43.07 DM: 3.7% HTN: 27.8% |  |  |  | n: 83 Age: 47 (30-67) Female: 59 (71.1) BMI: 44.12 (29.73-64.46) DM: 20.48% HTN: 50.60% |  |
| Fiorillo, 2020 | Obs / N | Single | Yes | n: 23 Age: 41 (35-43) Female: 16 (30.4) BMI: 39.5 (36.7-44.7) DM: 2 (8.7) HTN: 3 (13) |  |  |  | n: 23 Age: 37 (25-43) Female: 17 (73.9) BMI: 41 (38.3-43.4) DM: 3 (13) HTN: 7 (30.4) |  |
| Gomez, 2016 | RCT / Y | Single | No |  | n: 15 Age: 38.1 (8.8) Race White: 60%  Female: 87% BMI: 34.7 (3.42) |  |  |  | n: 14 Age: 38.2 (8.78) Race White: 85.7% Female: 93% BMI: 35.6 (2.84) |
| Lopez-Nava, 2021 | Obs / N | Multi | No | n: 199 Age: 44.6 (10) Female: 141 (71) BMI: 39.4 (5.4) |  |  |  | n: 61 Age: 44.6 (11.2) Female: 36 (59) BMI: 40.1 (3.7) |  |
| Novikov, 2018 | Obs / Y | Single | No | n: 91 Age: 43.86 (11.26) Female: 62 (68.13) BMI: 38.61 (6.98) DM: 20 (21.98) A1c: 5.82 (0.98) HTN: 18 (19.78) |  |  |  | n: 120 Age: 40.71 (11.95) Female: 94 (78.33) BMI: 47.22 (7.84) DM: 31 (25.83) A1c: 6.3 (1.25) HTN: 61 (50.83) |  |
| Sullivan, 2018 | RCT / Y | Multi | No |  | n: 198 Age: 42.7 (9.6) Race White: 165 (83.3) Female: 171 (86.4) BMI: 35.2 (2.7) A1c: 5.3 (0.4) HTN: 31 (15.7) |  |  |  | n: 189 Age: 42.5 (9.3) Race White: 155 (82.0) Female: 170 (89.9) BMI: 35.5 (2.7) A1c: 5.3 (0.5) HTN: 28 (14.8) |
| Sullivan, 2017 | RCT / Y | Multi | No | n: 221 Age: 44.2 (8.6) Race White: 154 (71) Black: 61 (28.1) Female: 195 (88.2) BMI: 36.0 (2.4)  EBW: 99.7 (12.2kg) DM: 17 (7.7) HTN: 100 (45.2) |  |  |  |  | n: 111 Age: 45.3 (9.1) Race White: 70 (64.8) Black: 34 (31.5) Female: 101 (91) BMI: 36.2 (2.2)  EBW: 98.7 (11.6kg) DM: 11 (9.9) HTN: 42 (37.8) |
| Thompson, 2017 | RCT / Y | Multi | No |  |  | n: 111 Age: 42.4 (10.0) Race White: 63 (56.8) Black: 33 (29.7) Hispanic: 11 (9.9)  Female: 96 (86.5) BMI: 42.0 (5.1) DM: 3 (2.7) A1c: 5.7 (0.6) HTN: 46 (41.4) |  |  | n: 60 Age: 46.8 (11.6) Race White: 31 (51.7) Black: 17 (28.3) Hispanic: 11 (18.3)  Female: 53 (88.3) BMI: 40.9 (3.9) DM: 8 (13.3) A1c: 5.8 (0.6) HTN: 24 (40.0) |
| Thompson, 2018 | RCT / Y | Multi | No |  |  | n: 111 Age: 42.4 (10.0) Race White: 63 (56.8) Black: 33 (29.7) Hispanic: 11 (9.9)  Female: 96 (86.5) BMI: 42.0 (5.1) DM: 3 (2.7) A1c: 5.7 (0.6) HTN: 46 (41.4) |  |  | n: 60 Age: 46.8 (11.6) Race White: 31 (51.7) Black: 17 (28.3) Hispanic: 11 (18.3)  Female: 53 (88.3) BMI: 40.9 (3.9) DM: 8 (13.3) A1c: 5.8 (0.6) HTN: 24 (40.0) |
| Lopez-Nava, 2020 | Obs / N | Multi | No | n: 12 Age: 49.3 (2.4) Female: 9 (75) BMI: 38.3 (1.8) DM: 0 HTN: 2 (17) |  |  |  | n: 12 Age: 50.5 (1.9) Female: 9 (75)  BMI: 39.2 (1.5) DM: 0 HTN: 9 (75) |  |
| Raftopoulos, 2019 | Obs / N | Single | Yes |  | n: 58 Age: 43.2 (11.8) Female: 70.7% BMI: 36.7 (5.7) |  |  |  | n: 413 Age: 48.3 (12.4) Female: 85.9% BMI: 36.8 (5.0) |
| Raftopoulos, 2019 | Obs / N | Single | Yes |  | n: 79 Age: 43 (10.8) Female: 68.4% BMI: 36.2 (5.4) |  |  |  | n: 413 Age: 48.3 (12.4) Female: 85.9% BMI: 36.8 (5.0) |
| Abu Dayyeh, 2019 | RCT / Y | Multi | No |  |  |  |  |  |  |
| Wilson, 2018 | Obs / N | Single | No |  |  | Unknown | Unknown |  |  |
| Sadek, 2017 | Obs / Y | Single | Unclear | n: 23 |  |  |  | n: 277 |  |
| Abd El Mohsen, 2017 | Obs / Y | Multi | No | n: 5 |  |  |  |  | n: 14 |
| Abu Dayyeh, 2015 | RCT / Y | Multi | No |  | n: 137 Age: 38.7 (9.4) Female: (89.6) BMI: 35.2 (3.17) EBW: 28.4 (2.7) |  |  |  | n: 136 Age: 40.8 (9.6) Female: (90) BMI: 35.4 (2.7) EBW: 28.7 (8.1) kg |
|  |  |  |  | ESG | Balloon | AspireAssist | RYGB | LSG | Lifestyle |
| Fuller, 2010 | RCT / N | Single | No |  | n: 31  Age: 43  Female: (68)  BMI: 36.0 |  |  |  | n: 35  Age: 48  BMI: 36.7 |
| Ponce, 2012 | RCT / Y | Multi | No |  | n: 21  Age: 38.9 (9.1)  White: (95)  Female: (81)  BMI: 34.7 (2.6) |  |  |  | n: 9  Age: 45.3 (6.6)  White: (100)  Female: (100)  BMI: 35.6 (2.0) |
| Lee, 2012 | RCT / N | Single | No |  | n: 8  Age: 43 (19.75)  Female: 5 (62.5)  BMI: 30.3 (4.22)  Diabetes: 1 (12.5)  NAFLD: 8 (100) |  |  |  | n: 10  Age: 47 (15)  Female: 2 (20)  BMI: 32.4 (6.66)  Diabetes: 1 (10)  NAFLD: 10 (100) |
| Ponce, 2015 | RCT / Y | Multi | No |  | n: 187  Age: 43.8 (9.5)  White: (81.8)  Black: (13.4)  Hispanic: (8)  Female: (95.2)  BMI: 35.3 (2.8)  Diabetes: (7)  HbA1c: 5.7 (0.7)  HTN: (28.9) |  |  |  | n: 139  Age: 44.0 (10.2)  White: (85.6)  Black: (11.5)  Hispanic: (5.8)  Female: (95.0)  BMI: 35.4 (2.6)  Diabetes: (7.2)  HbA1c: 5.7 (0.88)  HTN: (35.3) |
| Mohammed, 2014 | RCT / N | Single | No |  | n: 84  Age: 43.96 (8.98)  Female: (54)  BMI: 47.87 (1.08)  EBW: 65.45 (5.04) |  |  |  | n: 44  Age: 42.65 (6.61)  Female: (59)  BMI: 47.46 (1.85)  EBW: 65.23 (6.77) |
| Ahmed, 2019 | Obs / N | Single | No |  | n: 40  Female: 40 (100)  BMI: 36 |  |  |  | n: 40  Female: 40 (100)  BMI: 36.5 |
| Sullivan, 2012 | RCT / Y | Single | No |  |  | n: 11  BMI: 42 (4.7) |  |  | n: 7  BMI: 43.4 (5.3) |
| Salomone, 2021 | Obs / N | Single | No |  | n: 26  Age: 53  Female: (31)  Diabetes: (38)  HbA1c: 7.5  HTN: (65)  NAFLD: 26 (100) |  |  |  |  |
| Chan, 2021 | RCT / N | Multi | No |  | n: 26  Age: 38.1 (7.9)  Female (70)  BMI: 30.2 (2.3) |  |  |  | n: 23  Age: 35.3 (7.2)  Female: (75.5)  BMI: 30.2 (2.1) |
| Abeid, 2019 | Obs / N | Single | No |  | n: 1600  Age: 34.1 (10.3)  Female: (77)  BMI: 40.3 (8.17)  Diabetes: (6.8)  HTN: (15.06) |  |  |  |  |
| Alqahani, 2019 | Obs / N | Single | No | n: 1000  Age: 34.4 (9.5)  Female: (89.7)  BMI: 33.3 (4.5)  Diabetes: (1.7)  HTN: (2.8) |  |  |  |  |  |
| Mathus, 2014 | Obs / N | Single | No |  | n: 815  Age: 36.5 (9.8)  Diabetes: (2.3)  HTN: (15.6) |  |  |  |  |
| Sander, 2017 | Obs / N | Single | No |  | n: 9763  Age: 31.13  Female: (78)  BMI: 33.42 |  |  |  |  |
| Benias, 2020 | Obs / N | Single | No | n: 14  Age: 39 (4.2) |  |  |  | n: 11  Age: 47 (3.9) |  |
| Fuller, 2019 | RCT / Y | Single | No |  | n: 37  Age: 43.3 (9.4)  White: (83.9)  Female: (68)  BMI: 36 (2.7) |  |  |  | n: 37  Age: 48.1 (7.3)  White: (74.3)  Female: (66)  BMI: 36.9 (2.7) |
| Abu Dayyeh, 2021 | RCT / Y | Single | No |  | n: 187  Age: 44.4 (8.9)  White: 132 (71)  Black: 49 (26)  Asian: 1 (1)  Female: 162 (87)  BMI: 35.8 (2.6)  Diabetes: 13 (7)  HTN: 41 (22) |  |  |  | n: 101  Age: 44.0 (8.9)  White: 72 (71)  Black: 26 (26)  Asian: 1 (1)  Female: 90 (89)  BMI: 35.8 (2.7)  Diabetes: 4 (4)  HTN: 32 (32) |
| Moore, 2019 | Obs / Y | Multi | No |  | n: 1343  Age: 45.7 (10.8)  White: 897 (66.8)  Female: 1055 (78.6)  BMI: 35.4 (5.4) |  |  |  |  |
